# CPEB4-Promoted Paclitaxel Resistance in Ovarian Cancer *In Vitro* Relies on Translational Regulation of CSAG2

**DOI:** 10.3389/fphar.2020.600994

**Published:** 2021-01-13

**Authors:** Yaqing Zhang, Hongyun Gan, Fei Zhao, Xiaomei Ma, Xiaofeng Xie, Rui Huang, Jin Zhao

**Affiliations:** ^1^ Medical College of Northwest Minzu University, Lanzhou, China; ^2^ Department of Gynecology, Gansu Provincial People's Hospital, Lanzhou, China

**Keywords:** CPEB4, paclitaxel, resistance, ovarian carcinoma, CSAG2

## Abstract

**Background:** Drug resistance is a major obstacle in chemotherapy for ovarian cancer, wherein the up regulation of drug-resistant genes plays an important role. The cytoplasmic polyadenylation element binding protein 4 (CPEB4) is an RNA binding protein that controls mRNA cytoplasmic polyadenylation and translation.

**Methods:** The expression of CPEB4 in paclitaxel-resistant ovarian cancer cell lines and recurrent ovarian tumors relative to counterparts was determined by qRT-PCR, Western blotting and immunohistochemistry. The response to paclitaxel treatment was evaluated by cellular viability test and colony formation assay. RNA immunoprecipitation and poly(A) tail test were applied to examine the levels of RNA binding and cytoplasmic polyadenylation.

**Results:** CPEB4 is elevated in paclitaxel-resistant ovarian cancer cells and recurrent ovarian tumors treated with paclitaxel-based chemotherapy. In addition, CPEB4 overexpression promotes paclitaxel resistance in ovarian cancer cells *in vitro*, and vice versa, CPEB4 knockdown restores paclitaxel sensitivity, indicating that CPEB4 confers paclitaxel resistance in ovarian cancer cells. Mechanistically, CPEB4 binds with the taxol (paclitaxel)-resistance-associated gene-3 (TRAG-3/CSAG2) mRNAs and induces its expression at a translational level. Moreover, CSAG2 expression is upregulated in paclitaxel-resistant ovarian carcinoma and cancer cell lines, and more importantly, siRNA-mediated CSAG2 knockdown overtly attenuates CPEB4-mediated paclitaxel resistance.

**Conclusion:** This study suggests that the drug-resistant protein CSAG2 is translationally induced by CPEB4, which underlies CPEB4-promoted paclitaxel resistance in ovarian cancer *in vitro*. Thus, interfering CPEB4/CSAG2 axis might be of benefit to overcome paclitaxel-resistant ovarian cancer.

## Introduction

Ovarian cancer is the most lethal gynecologic malignancy, and approximate 22,440 new cases and 14,080 deaths have been estimated to occur in the United States in 2017 (Siegel et al., 2017). The standard first-line option for treating ovarian cancer is cytoreductive surgery followed by paclitaxel/platinum-based chemotherapy (Jayson et al., 2014). Although up to 80% of patients initially respond well to therapy, most of them develop recurrent ovarian cancer within 12–24 months and succumb to progressive refractory disease resistant to therapy, with a poor 5-years survival rate lower than 30% (Au Yeung et al., 2016). Therefore, there is an urgent need to develop a new therapeutic strategy targeting mechanisms leading to chemoresistance.

The development of resistance to paclitaxel is multifactorial and several mechanisms have been documented, including enhanced activity of xenobiotics transporter P-glycoprotein (Alam et al., 2019), alterations of β-tubulin (Yang et al., 2016) and remodeling of tumor microenvironment (Au Yeung et al., 2016). Recently, CSAG2, also known as the taxol resistance associated gene 3 (TRAG-3), has been identified to be overexpressed in a paclitaxel-resistant ovarian cancer cell line and also be a prognostic factor for predicting clinical outcome after paclitaxel-based chemotherapy (Lage and Denkert, 2007; Materna et al., 2007). Whereas, the specific role of CSAG2 in paclitaxel resistance is still unknown.

The reprogramming of gene expression *via* posttranscriptional regulation of specific mRNA subpopulations is an important mechanism underlying the broad modulation of expression of genes responsible for governing the malignant properties (Wurth et al., 2016; Hu et al., 2020). This regulation is primarily mediated by common *cis*-acting elements present in 3′ untranslated regions (UTRs), such as the cytoplasmic polyadenylation element (CPE) (Chen Y. et al., 2016), which is bound by the CPE binding proteins (CPEBs) (Harvey et al., 2018). To date, CPEBs have been found to act as either translational repressors or activators to regulate cell cycle, energy metabolism and senescence (Kang et al., 2020). Moreover, CPEB4, one member of CPEBs, has been shown to play a key role in the progression of pancreatic ductal adenocarcinoma, glioblastoma, glioma and gastric cancer (Ortiz-Zapater et al., 2011; Zhijun et al., 2017; Cao et al., 2018). In this study, we report that CPEB4 promotes paclitaxel resistance in ovarian cancer, and suggest that this effect depends on its translational regulation of CSAG2.

## Materials and Methods

### Antibodies and Reagents

The antibodies and paclitaxel were purchased from the following sources: CPEB4 (Novus, NBP2-15984), CSAG2 (Invitrogen, PA5-25339), β-actin (Santa Cruz, sc-69879), goat anti-mouse IgG-HRP (Santa Cruz, sc-2302), goat anti-rabbit IgG-HRP (Santa Cruz, sc-2004), goat anti-rabbit peroxidase-conjugated IgG (Sigma-Aldrich, A0545), and paclitaxel (Sigma-Aldrich, T7191).

### Ovarian Tumor Specimens and Immunohistochemistry

Eighteen formalin-fixed and paraffin-embedded ovarian cancer specimens were obtained from Gansu Provincial Hospital under the ethical approval by the Ethics Committee of Medical College of Northwest Minzu University. Informed written consent was obtained from all patients, whose detailed clinical information was shown in Supplementary Table S1. Immunohistochemistry was performed to compare the expression levels of CPEB4 or CSAG2 in paired primary tumors before treatment and recurrent ovarian tumors post-chemotherapy, who received standard paclitaxel-based chemotherapy for three to six courses of treatment. Briefly, 5 μm specimen sections were prepared. Antigen retrieval was conducted by immersing slides in 0.1 M citrate (pH 6.0) at 120°C for 15 min. Slides were blocked with 5% goat serum for 1 h at room temperature (RT) prior to incubation with primary antibodies for overnight at 4°C. The isotype IgG antibody was used as negative control. After incubation, peroxidase-conjugated secondary antibodies were added onto slides for further 1 h incubation at RT. Eventual reactions were performed using diaminobenzidine (DAB) as chromogenic substrate [EnVision + System (DAKO)]. At last, sections were counterstained with haematoxylin, and visualized under a Leica DM6000 Digital microscope, and images were acquired using QWin software (Leica). The immunoreactivity was scored by the H score system (Wallin et al., 2016), ranging from zero to three, as assessed by two investigators based on the percentage of stained cells and intensity of staining.

### Cell Culture and Generation of Paclitaxel-Resistant Cells

Ovarian carcinoma cell lines SKOV3 and CaOV3, and human embryonic kidney 293T (HEK293T) cell line were obtained from the American Tissue Culture Center (Manassas, VA). All cell lines were cultured in a humidified incubator (ThermoFisher Scientific) at 37°C with 5% CO_2_. SKOV3 and CaOV3 cell lines were maintained in complete RPMI-1640 medium (ThermoFisher Scientific) with 10% fetal bovine serum (FBS). HEK293T cell line was maintained in complete dulbecco’s modified eagle’s medium (DMEM) (ThermoFisher Scientific) with 10% FBS. The paclitaxel-resistant ovarian cancer cell lines SKOV3 (R) and CaOV3 (R) were established by over 7-months culture with increasing concentrations of paclitaxel, resulting in approximately 6.3-fold and 7.0-fold as compared with the parental cell lines, respectively. In brief, paclitaxel-resistant SKOV3 (R) and CaOV3 (R) were selected by culturing cells with paclitaxel in a dose-escalation manner using 72-h exposure. SKOV3 and CaOV3 cells were initially cultured with 5 nM paclitaxel. When sensitive cells were removed and surviving SKOV3 and CaOV3 cells repopulated the flask, the concentration of paclitaxel was increased to 10, 25, 50, 100, and 200 nM. After 7 and 8 months, the paclitaxel-resistant SKOV3 and CaOV3 cells were obtained, respectively. Meanwhile, naïve SKOV3 and CaOV3 cells were cultured with DMSO in the same manner. After resistance testing *via* continuous 1-week culture with 200 nM paclitaxel, the resistant SKOV3 and CaOV3 cells were maintained in the presence of 10 nM paclitaxel, as compared with DMSO for naïve cells.

### Cellular Viability Test

Cells were harvested, then immersed in PBS, and stained with 0.4% trypan blue solution (Sigma-Aldrich, T8154). Trypan blue positive cells were defined as nonviable cells and excluded from the counting. The number of trypan blue negative cells were determined by TC20 Automated Cell Counter (Bio-Rad), and its percentage (%) within total cell number was calculated.

### Colony Formation Assay

Cells were harvested and counted by TC20 Automated Cell Counter as described before. A total of 1,000 viable cancer cells in each group were seeded in six-well plate and cultured with 2 ml medium. Three replicates were set for each treatment condition. The culture medium with or without paclitaxel was replaced with fresh medium every 3 days until the formed colonies were clearly visible for naked eyes. Eventually, colonies were washed with PBS, fixed with 4% paraformaldehyde solution (Santa Cruz, sc-281692), and stained by 0.05% crystal violet (Sigma-Aldrich, V5265). The final images were photographed and the ImageJ software was used to analyze the number of colonies in each well.

### Protein Extraction and Western Blotting

The whole cell proteins were extracted from cells lyzed in RIPA lysis buffer (Beyotime, P0013C) with protease inhibitor Cocktail (Roche, CO-RO) on ice for 10 min. The lysates were centrifuged with 12,000 × *g* at 4°C for 20 min. Protein samples in supernatants were denatured with 5× loading buffer at 100°C for 10 min. Equal amount of proteins were resolved by 10% SDS-PAGE, transferred onto Immobilon-P PVDF membrane (Millipore, IPVH00010), and then blocked by 5% bovine serum albumin (BSA) (Roche) saluted in tris buffered saline (TBS) supplemented with 0.1% Tween (TBST) for 1 h at RT. PVDF membrane were then incubated with primary antibodies overnight at 4°C. Next, membranes were washed with TBST for three times, and incubated further with secondary antibodies at RT for 1 h. Membranes were again washed with TBST for three times and reacted with the enhanced chemiluminescence (GE Healthcare, RPN2209) for protein detection under GE ImageQuant LAS 4000 instrument. The intensity of protein bands was quantified with ImageJ software.

### RNA Extraction and qRT-PCR Analysis

Cells were harvested and homogenized, and the total RNA were extracted with agents according to Trizol-based method (Thermo Fisher, 15596026). Procedures were performed as described in the manufacturer’s instructions. The levels of target transcripts were determined by SYBR green real-time PCR kit (TakaRa, RR420A) and run by the 7500 Real-Time PCR System (Applied Biosystems). Data were analyzed based on the comparative Ct method. Human *ACTB* was used as an endogenous control. Primer sequences are listed as follows: CPEB4 sense 5′-TGG​GGA​TCA​GCC​TCT​TCA​TA-3′, antisense 5′-CAA​TCC​GCC​TAC​AAA​CAC​CT-3′; CSAG2 sense 5′-CAA​CAT​CTC​TGC​CGC​TAA​CC-3′, antisense 5′-GTA​GCC​GCC​GAC​ATT​ACA​AG-3′. ACTB sense 5′-ACG​GGC​ATT​GTG​ATG​GAC​TC-3′, antisense 5′-GTG​GTG​GTG​AAG​CTG​TAG​CC-3′.

### Overexpression *via* Retrovirus Infection

Clone of human CPEB4 was constructed into pBABE-puro system (addgene, 1764), and empty vector construct was used as a control. Retrovirus expressing these retrovirus plasmids were packaged with HEK293T cells. Cancer cells were cultured and infected with retrovirus solution in the presence of 4 μg/ml polybrene for 18°h, and further cultured for 1°day in fresh medium. Cells infected with retrovirus were selected by culturing with 2 μg/ml puromycin for 1–2 weeks. The stably infected cells were maintained in culture medium containing 1 μg/ml puromycin. Overexpression was confirmed by qRT-PCR or Western blotting analysis.

### shRNA and siRNA-Mediated Knockdown

shRNA-mediated knockdown of human CPEB4 was implemented using a specific targeting sequence (5′-CCG​GGC​CTG​CCT​CAT​TTG​GCG​AAT​ATT​TCT​CGA​G AAT​ATT​CGC​CAA​ATG​AGG​CAG​GCT​TTT​TG-3′). A non-specific ‘scrambled’ shRNA was used as a control. These sequences were constructed into the pLKO.1-puro vector (Sigma, SHC201). For producing lentivirus, plasmids carrying these sequences were co-transfected with the packaging plasmids into HEK293T cells. At 2 days following transfection, supernatants containing lentivirus were harvested and stored at −80°C if not being used immediately. The cultured cancer cells were then infected with lentivirus solution for 18 h in the presence of 4 μg/ml polybrene. The stably infected cells were obtained by culturing them under the selective pressure of 2 μg/ml puromycin for 1–2 weeks. For gene knockdown mediated by siRNA transfection, cancer cells were transfected with 20 nM siRNAs targeting luciferase control (siCtrl), *CSAG2* (siCSAG2) using Lipofectamine RNAimax (Invitrogen, 13778150) for 24 h. Medium was replaced with fresh medium and cells were cultured for further 2 days. Gene knockdown was confirmed by qRT-PCR or Western blotting analysis.

### RNA Immunoprecipitation

RNA immunoprecipitation (RIP) was performed using a MagnaRIP Kit (Millipore) as described previously (Lu et al., 2016). Briefly, Cells were harvested and lyzed with RIP lysis buffer (10 mM HEPES, pH 7.0, 100 mM KCl, 5 mM MgCl2, 0.5% NP-40, 1 mM DTT) for 20 min on ice. After centrifuge, the whole cell lysates were incubated at 4°C overnight with magnetic protein A-protein G beads (Sigma-Aldrich) coupled with isotype IgG control or CPEB4 antibody to obtain RNA-protein immunocomplexes. Beads were washed three times with washing buffer, and incubated with proteinase K buffer for 45 min at constant 55°C, which was followed by RNA isolation from the immunoprecipitates according to the manufacturer’s instructions. cDNA was reversely transcripted by First-strand cDNA Synthesis System (Thermo Scientific, K1621). qRT-PCR was performed by amplifying a 300-bp region in the 3′ UTR of each transcript.

### Poly(A) Tail Test

Poly(A) tail test was performed as described previously (Pique et al., 2008; Ortiz-Zapater et al., 2011). Briefly, 3 μg of total RNA were ligated to sense anchor primer (5′P-GGTCACCTCTGATCTGGAAGCGAC-NH_2_-3′) and reversely transcribed with antisense anchor primer (GTC​GCT​TCC​AGA​TCA​GAG​GTG​ACC​TTT​TT). The product was then treated with 2 μg RNAse A. This final product was used as a template for PCR amplification with antisense anchor primer and a specific primer for each mRNA analyzed. FastStart Taq DNA polymerase (Roche, FPCRN-RO) was utilized as recommended by the manufacturer in the absence or presence of 5 μCi (^32^P) α-dATP. PCR products were separated in a denaturing 8% polyacrylamide gel and visualized by autoradiography.

### Statistical Analyses

Statistical analyses were performed with GraphPad Prism 6. Data are presented as mean ± SEM. Statistical analyses comparing data between two groups were assessed by unpaired Student’s *t* test, and ANOVA with a *post hoc* Dunnett’s test was used for analyzing data among more than two groups. *p* < 0.01 and *p* < 0.05 indicate a statistical difference.

## Results

### CPEB4 Is Upregulated in Paclitaxel-Resistant Ovarian Cancer Cells and Recurrent Ovarian Tumors

**FIGURE 1:**
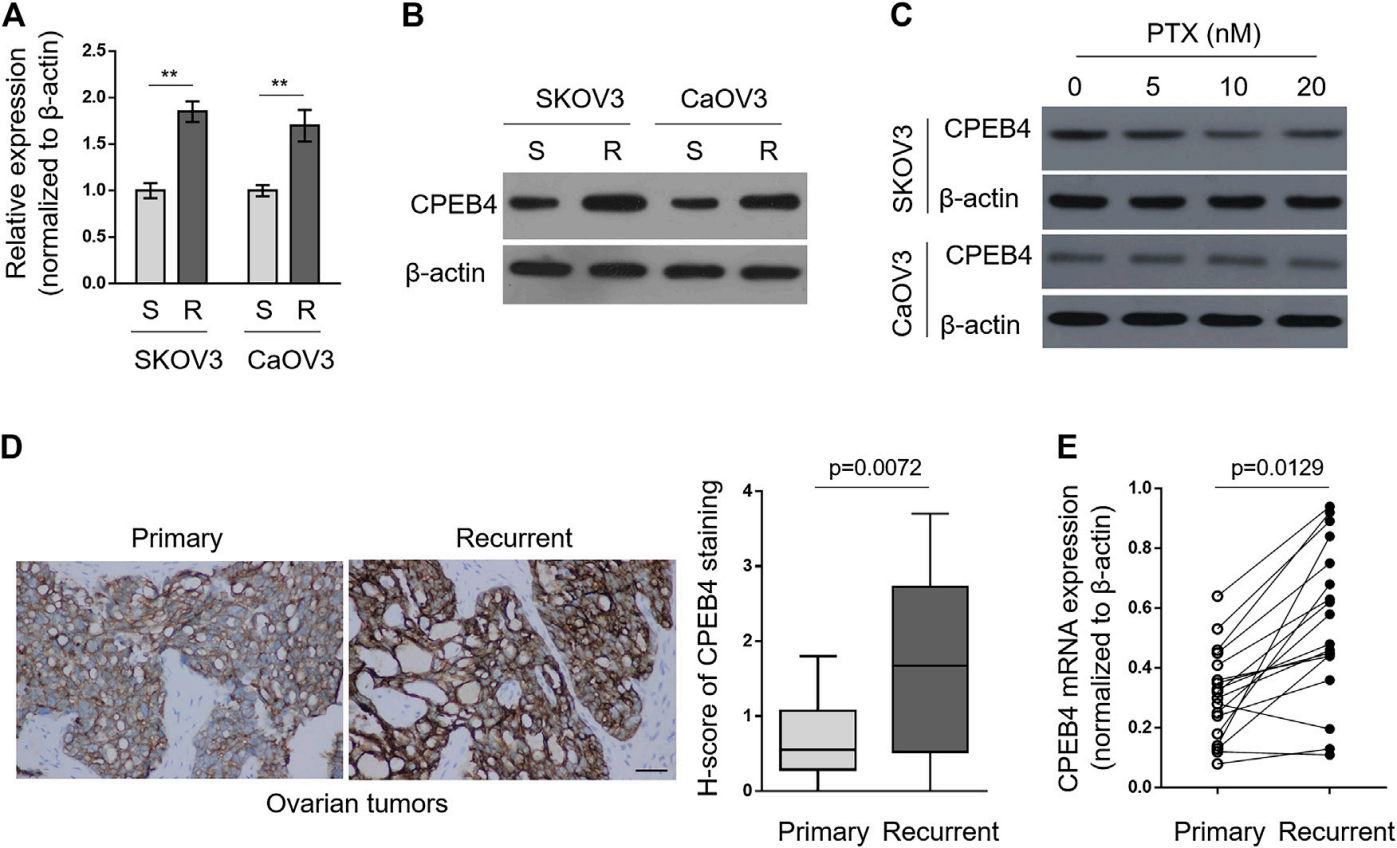
CPEB4 is upregulated in paclitaxel-resistant ovarian cancer cells and recurrent ovarian tumors. **(A,B)** The paclitaxel-resistant ovarian cancer cell lines SKOV3 (R) and CaOV3 (R) were established with treatment of increasing concentrations of paclitaxel (PTX) for over 7 months. The mRNA **(A)** and protein **(B)** levels of CPEB4 in these cells were analyzed by qRT-PCR and Western blotting analysis, and compared with those of naive sensitive cells, SKOV3 (S) and CaOV3 (S). β-Actin was used as a loading and reference control. The representative images are shown. Data represent mean ± SEM. *n* = 3. **p < 0.01. **(C)** SKOV3 **(upper)** and CaOV3 **(lower)** naive cells were treated with indicated concentrations of PTX for 72 h. The protein level of CPEB4 was analyzed by Western blotting. β-Actin was used as a loading control. The representative images are shown. **(D)** Representative images **(left)** of immunohistochemical staining of CPEB4 from matched primary and recurrent ovarian tumors treated with paclitaxel-based chemotherapy. Scale bar, 50 µm. H-score **(right)** is used to semi-quantify CPEB4 expression levels. The black line inside the box is the median, and the lines above and below the box indicate the maximum and minimum of the H-scores. Each group contained 18 paired samples. **(E)** The mRNA levels of CPEB4 in matched primary and recurrent ovarian tumors **(D)** were analyzed by qRT-PCR. β-Actin was used as a reference control. Each symbol represents the mean value of three replicates.

To explore whether CPEB4 plays a role in paclitaxel resistance in ovarian cancer, we compared its transcript levels between naive sensitive SKOV3 (S) cells and their paclitaxel-resistant counterparts SKOV3 (R) through qRT-PCR analysis. As depicted, the transcript level of CPEB4 in SKOV3 (R) was nearly twice than that of SKOV3 (S) (Figure 1A, left half). Additionally, similar tendency was obtained when comparing another pair of ovarian cancer cell lines, CaOV3 (S) and CaOV3 (R) (Figure 1A, right half). We next examined the protein level of CPEB4 by performing Western blotting analysis. As shown in Figure 1B, in consistent with its transcript level, the protein level of CPEB4 was upregulated in both SKOV3 (R) and CaOV3 (R), as compared with their naive sensitive counterparts. Of note, we further found that CPEB4 failed to be increased in SKOV3 (S) and CaOV3 (S) treated with paclitaxel for 72 h (Figure 1C). Overall, these results suggest that the upregulation of CPEB4 was associated with functional resistance of ovarian cancer cells to paclitaxel and is impossibly due to acute inducible effect of paclitaxel in naive sensitive cells.

Next, we performed immunohistochemistry to compare CPEB4 expression between paired primary untreated ovarian tumors and recurrent tumors treated with paclitaxel-based chemotherapy. The result showed that CPEB4 had stronger immunoreactive staining in recurrent tumors than that of primary tumors (Figure 1D, left), and semi-quantification using H-score analysis proved the higher expression level of CPEB4 in recurrent ovarian tumors (Figure 1D, right). The transcript level of CPEB4 in these paired tumors was also quantified, and likewise, significant upregulation of CPEB4 was observed in recurrent ovarian tumors (Figure 1E). Collectively, these data indicate that CPEB4 is upregulated in paclitaxel-resistant ovarian cancer cells and recurrent ovarian tumors, and also imply an association of its upregulation with paclitaxel resistance.

### CPEB4 Promotes Paclitaxel Resistance in Ovarian Cancer Cells *In Vitro*

**FIGURE 2:**
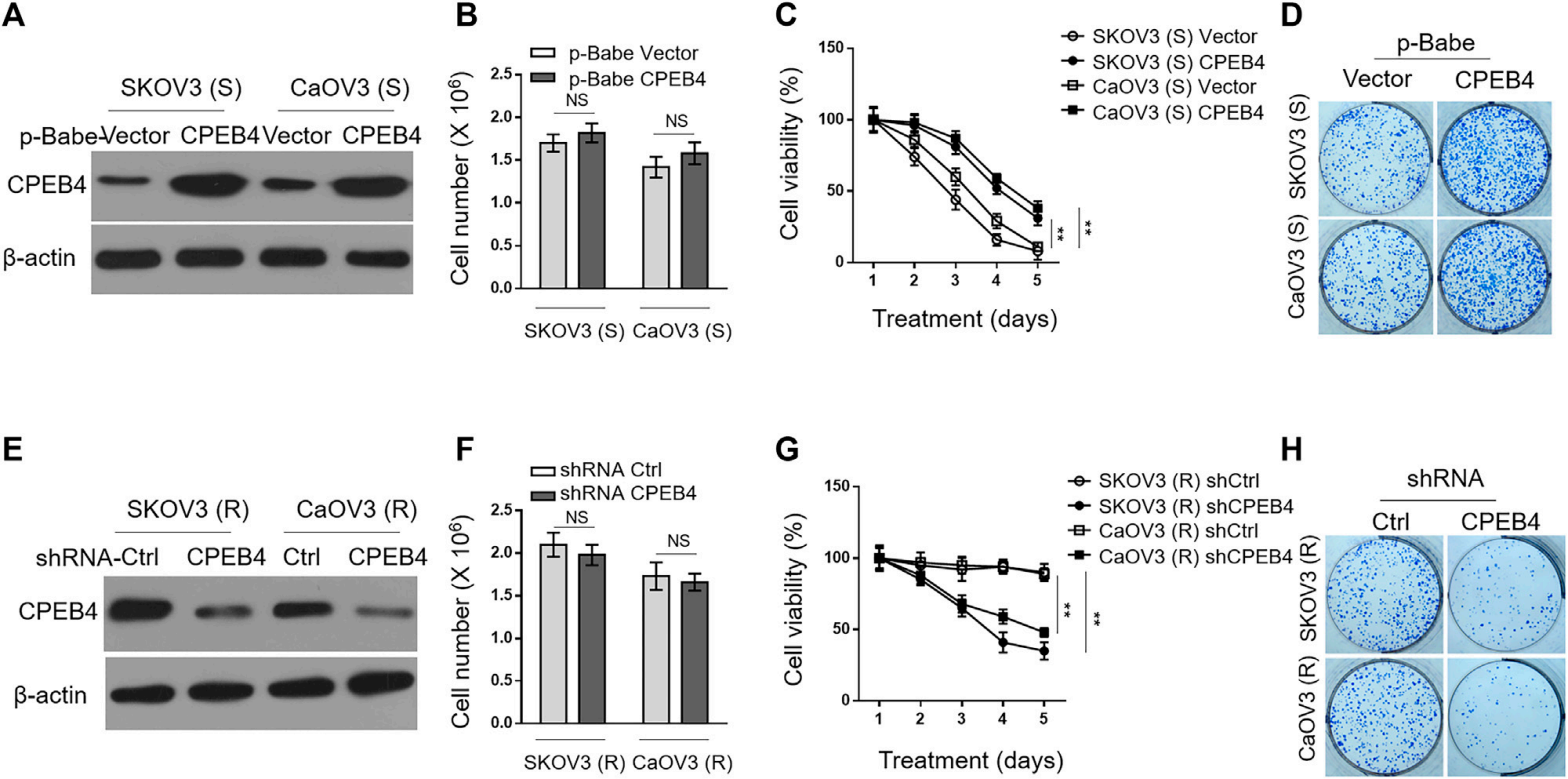
CPEB4 promotes paclitaxel resistance in ovarian cancer cells *in vitro*. **(A)** The naive sensitive cells, SKOV3 (S) and CaOV3 (S), were stably infected with retrovirus expressing empty vector or human CPEB4, and the protein level of CPEB4 was analyzed by Western blotting. The representative images are shown. **(B)** The viable cell numbers of cells **(A)** cultured for 3 days with an initial 2 × 10^6^ cell number. Trypan blue staining was used to exclude unviable cells. Data represent mean ± SEM. *n* = 3. NS, not significant. **(C)** Cells **(A)** were cultured with an initial 2 × 10^6^ cell number and then treated with 20 nM PTX for indicated periods of time. The viable cell number relative to DMSO treated group is shown. Data represent mean ± SEM. *n* = 3. **p < 0.01. **(D)** Representative images of cells **(A)** stained with crystal violet after 2-weeks culture with 20 nM PTX treatment **(left)**. The colony number was quantified by ImageJ **(right)**, and results relative to vector are shown. Data are mean ± SEM. *n* = 3. **p < 0.01. **(E)** The paclitaxel-resistant cells, SKOV3 (R) and CaOV3 (R), were stably infected with lentivirus expressing shRNA targeting control or human CPEB4, and the protein level of CPEB4 was analyzed by Western blotting. The representative images are shown. **(F)** The viable cell numbers of cells **(E)** cultured for 3 days with an initial 2 × 10^6^ cell number. Trypan blue staining was used to exclude unviable cells. Data represent mean ± SEM. *n* = 3. NS, not significant. **(G)** Cells **(E)** were cultured with an initial 2 × 10^6^ cell number and then treated with 20 nM PTX for indicated periods of time. The viable cell number relative to DMSO treated group is shown. Data represent mean ± SEM. *n* = 3. **p < 0.01. **(H)** Representative images of cells **(E)** stained with crystal violet after 2-weeks culture with 20 nM PTX treatment **(left)**. The colony number was quantified by ImageJ **(right)**, and results relative to control are shown. Data are mean ± SEM. *n* = 3. **p < 0.01.

To understand the effect of CPEB4 upregulation on paclitaxel resistance, we enforced its expression in SKOV3 (S) and CaOV3 (S) *via* retrovirus infection (Figure 2A). The stable overexpression of CPEB4 did not obviously affect cell proliferation or survival (Figure 2B), however, it markedly increased the survival rate of both two ovarian cancer cell lines when treated with paclitaxel (Figure 2C). In addition, higher colony formation rate was also found in CPEB4-overexpressing cells compared with vector controls, in the presence of continuous paclitaxel exposure (Figure 2D). Therefore, these results support a promotive effect of CPEB4 overexpression on paclitaxel resistance in ovarian cancer cells, and also suggest that CPEB4 upregulation detected in paclitaxel-resistant cells (Figures 1A,B) may contribute to the development and maintenance of resistance. To test this idea, we performed shRNA-mediated CPEB4 stable knockdown in SKOV3 (R) and CaOV3 (R) cells (Figure 2E). Under untreated condition, CPEB4 knockdown had no obvious effect on cell proliferation or survival (Figure 2F). But, when cells were continuously treated with paclitaxel, CPEB4-deficient cells exhibited lower rates of survival (Figure 2G) and colony formation (Figure 2H) compared with shCtrl group, indicating that CPEB4 is essential for paclitaxel-resistant property. Together, these lines of evidence prove that CPEB4 upregulation promotes paclitaxel resistance in ovarian cancer cells *in vitro*.

### CPEB4 Binds With CSAG2 mRNA and Induces Its Cytoplasmic Polyadenylation and Translation

**FIGURE 3:**
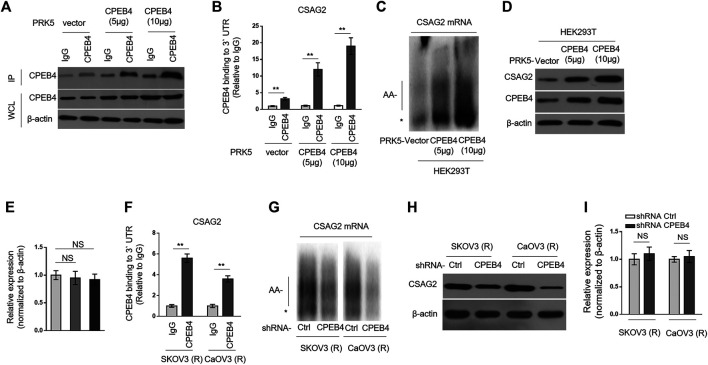
CPEB4 binds with CSAG2 mRNAs and induces its cytoplasmic polyadenylation and translation **(A,B)** HEK293T cells were transfected with 5 or 10 µg plasmids expressing vector or human CPEB4 as indicated, and the cell lysates were immunoprecipitated with IgG isotype control antibody or CPEB4 antibody. The protein level of CPEB4 in whole cell lysates and IP products was analyzed by Western blotting **(A)**. Total RNAs bound with agarose beads were purified from immunoprecipitates and reversely transcribed to cDNA and analyzed by qRT-PCR to measure the level of CSAG2 transcript **(B)**. The results in each sample represent the mean value of three replicates. The enrichment value relative to IgG group is shown. Data are mean ± SEM. **p < 0.01. **(C–E)** HEK293T cells were transfected with 5 or 10 µg plasmids expressing vector or human CPEB4 as indicated and cultured for 3 days. **(C)** Total RNAs were then extracted and the polyadenylation was measured by RNA ligation-coupled RT-PCR using (^32^P) α-dATP and specific primers for CSAG2. Products were separated in a denaturing 8% polyacrylamide gel and visualized by autoradiography. *, non-adenylated RNAs; AA-, adenylated RNAs. **(D)** The whole cell lysates were analyzed by Western blotting for detecting the level of CSAG2. β-Actin was used as a loading control. Shown here are representative images. **(E)** The mRNA of CSAG2 was analyzed by qRT-PCR. β-Actin was used as a reference control. Data are mean ± SEM. *n* = 3. NS, not significant. **(F)** The cell lysates of SKOV3 (R) and CaOV3 (R) cells were immunoprecipitated with IgG isotype control antibody or CPEB4 antibody. The binding of 3′-UTR of CSAG2 mRNA with CPEB4 was analyzed as in **(B)**. Data are mean ± SEM. **p < 0.01. **(G–I)** SKOV3 (R) and CaOV3 (R) cells stably expressing shRNA targeting control or human CPEB4 were routinely cultured for 3 days. The polyadenylation of CSAG2 mRNA **(G)**, and the protein level **(H)** and mRNA level **(I)** of CSAG2 were analyzed as in **(C–E)**. Data are mean ± SEM. *n* = 3. NS, not significant.

CSAG2, also known as taxol (paclitaxel)-resistance-associated gene-3 (TRAG-3), is overexpressed in paclitaxel-resistant ovarian cancer cells (Duan et al., 1999), and also shown to be negatively associated with clinical outcome after paclitaxel treatment (Lage and Denkert, 2007; Materna et al., 2007). These clues imply a role of CSAG2 in paclitaxel resistance. Moreover, in a genome-wide screening study, CSAG2 was found to be coimmunoprecipitated with CPEB4 in pancreatic ductal adenocarcinoma cells line (Ortiz-Zapater et al., 2011), although remains to be validated further. These prompt us to investigate whether CSAG2 mediates the effect of CPEB4 on paclitaxel resistance. Firstly, we validated the binding between CPEB4 and CSAG2 in HEK293 cells which were enforced to express different amounts of exogenous CPEB4 (Figure 3A). The results from RNA immunoprecipitation (RIP) showed that CPEB4 indeed bound with the 3′ UTR of CSAG2 mRNA, and the binding intensity was positively correlated with the amount of CPEB4 (Figures 3A,B). To assess whether the cytoplasmic polyadenylation of CSAG2 mRNA was regulated by CPEB4, we measured the length of poly(A) tail of CSAG2 mRNA by anchored RT-PCR (Pique et al., 2008). In CPEB4-overexpressing HEK293 cells, the poly(A) tail was elongated along with increased CPEB4 level, which is in contrast to the short poly(A) tail observed in control cells (Figure 3C), illustrating that the CSAG2 mRNA is more polyadenylated when CPEB4 is overexpressed. Cytoplasmic polyadenylation plays a key role in the translational control of mRNAs (Mendez and Richter, 2001). Consistent with elongated poly(A) tail, the protein level of CSAG2 was induced upon CPEB4 overexpression (Figure 3D), in parallel, with its transcript level unaffected (Figure 3E). Together, these data show that CSAG2 expression is regulated by CPEB4-mediated translational control. We further examined whether this mechanism also exists in ovarian cancer cells. RIP assay showed that CPEB4 also bound with CSAG2 mRNA in SKOV3 (R) and CaOV3 (R) cells (Figure 3F). Moreover, shRNA-mediated CPEB4 knockdown resulted in shorter poly(A) tail of CSAG2 mRNA in these cells (Figure 3G), and accordingly, CSAG2 expression was decreased (Figure 3H), and its transcript level remained unchanged (Figure 3I). Therefore, these findings indicate that CPEB4 can bind with CSAG2 mRNA and induce cytoplasmic polyadenylation and translationally control its expression.

### CSAG2 Is More Polyadenylated and Translated by CPEB4 in Paclitaxel-Resistant Ovarian Cancer Cells and Recurrent Ovarian Tumors

**FIGURE 4:**
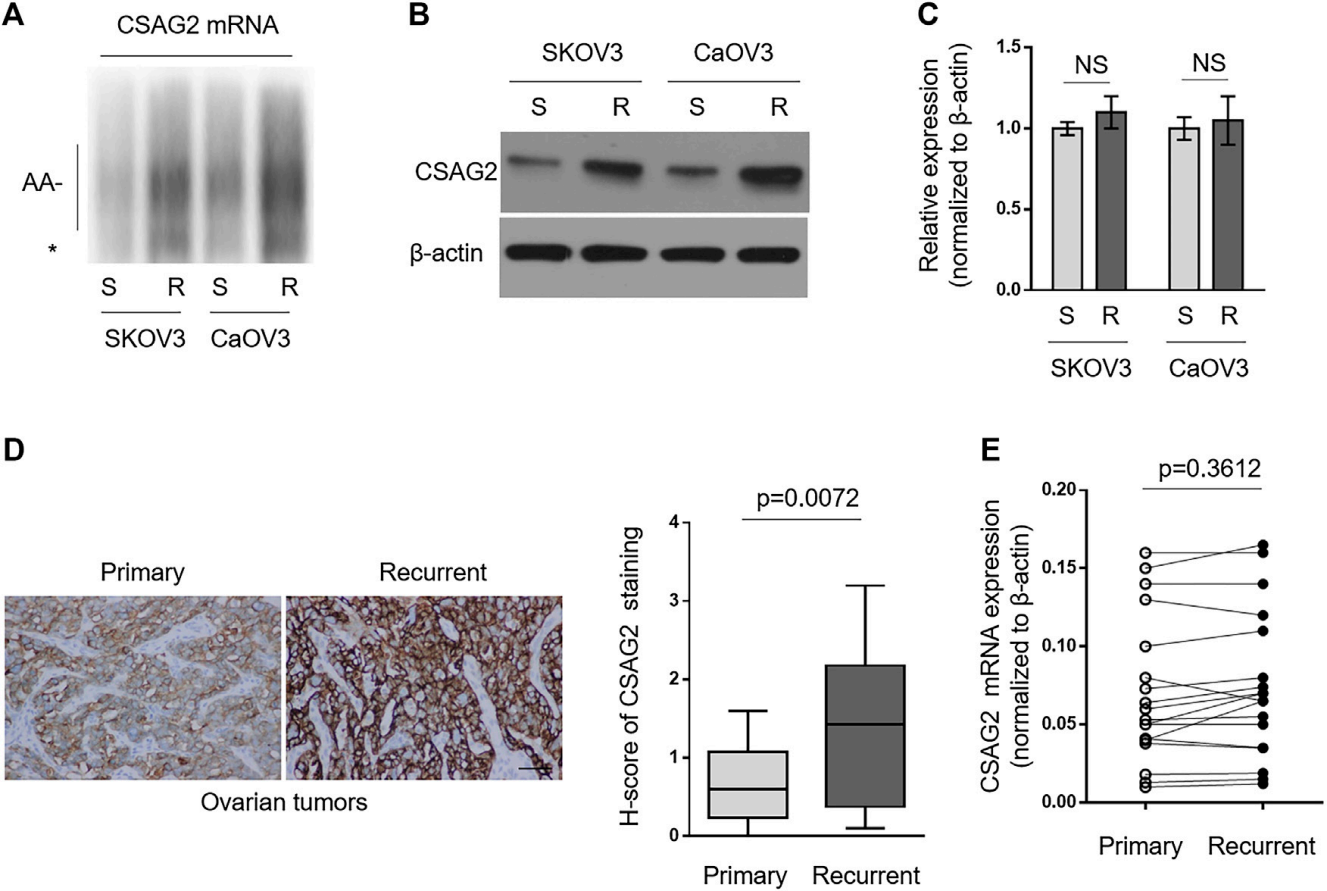
CSAG2 is more polyadenylated and translated by CPEB4 in paclitaxel-resistant ovarian cancer cells and recurrent ovarian tumors. **(A–C)** Cells of SKOV3 (R), SKOV3 (S), CaOV3 (S), CaOV3 (R) were routinely cultured for 3 days. **(A)** Total RNAs were extracted and the polyadenylation was assessed as described before. *, non-adenylated RNAs; AA-, adenylated RNAs. **(B)** The protein level of CSAG2 in cell lysates was analyzed by Western blotting. β-Actin was used as a loading control, and representative images are shown. **(C)** The mRNA of CSAG2 was analyzed by qRT-PCR. β-Actin was used as a reference control. Data are mean ± SEM. *n* = 3. NS, not significant. **(D)** Representative images **(left)** of immunohistochemical staining of CSAG2 from matched primary and recurrent ovarian tumors treated with paclitaxel-based chemotherapy. Scale bar, 50 µm. H-score **(right)** of CSAG2 staining is used to semi-quantify its expression levels. The black line inside the box is the median, and the lines above and below the box indicate the maximum and minimum of the H-scores. Each group contained 18 paired samples. **(E)** The mRNA levels of CPEB4 in matched primary and recurrent ovarian tumors **(D)** were analyzed by qRT-PCR. β-Actin was used as a reference control. Each symbol represents the mean value of three replicates.

Since paclitaxel-resistant ovarian cancer cells have higher expression level of CPEB4 (Figures 1A,B), together with the results mentioned above (Figure 3), we speculated that CSAG2 mRNA maybe more polyadenylated and expressed in paclitaxel-resistant ovarian cancer cells than that of naïve sensitive ones. As expected, anchored RT-PCR analysis revealed that poly(A) tail of CSAG2 mRNA was longer in KOV3 (R) and CaOV3 (R) cells (Figure 4A). Consistently, its protein level was increased (Figure 4B) and transcript level was not obviously affected (Figure 4C). Furthermore, keeping in line with these observations, CSAG2 showed stronger immunoreactive staining (Figure 4D, left) and higher H-score (Figure 4D, right) in recurrent tumors than that of primary tumors, and similarly, its transcript level did not show significant change (Figure 4E). Taken together, these data suggest that, due to different CPEB4 levels, CSAG2 expression is differentially regulated by CPEB4-mediated translational control in paclitaxel-resistant ovarian cancer cells and recurrent ovarian tumors compared with their counterparts, which leads to CSAG2 accumulation in resistant cells.

### CSAG2 Knockdown Attenuates CPEB4-Mediated Paclitaxel Resistance in Ovarian Cancer Cells

**FIGURE 5:**
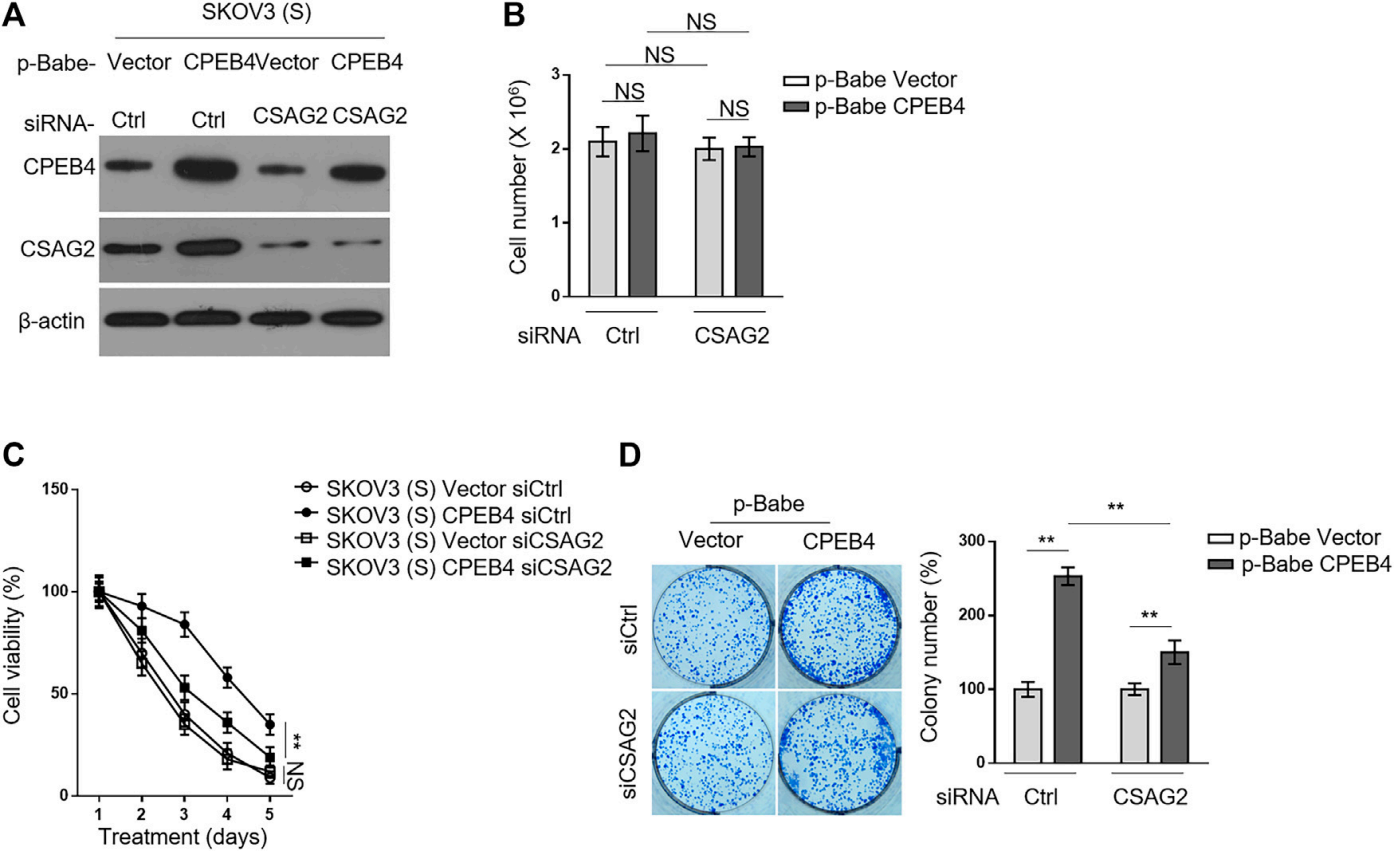
CSAG2 knockdown attenuates CPEB4-mediated paclitaxel resistance in ovarian cancer cells. **(A)** Cells of SKOV3 (S) and CaOV3 (S) stably expressing empty vector or human CPEB4 were transfected with siRNA targeting control or CSAG2, and further cultured for 3 days. The protein levels of were analyzed by Western blotting. β-Actin was used as a loading control. The representative images are shown. **(B)** Cells were treated as in **(A)**, and the viable cell number was counted by Trypan blue staining to exclude unviable cells. Data represent mean ± SEM. *n* = 3. NS, not significant. **(C)** Cells were treated as in **(A)**, and then treated with 20 nM PTX for indicated periods of time. The viable cell number relative to DMSO treated group is shown. Data represent mean ± SEM. *n* = 3. **p < 0.01; NS, not significant. **(D)** Cells were treated as in **(A)**, and then further cultured with 20 nM PTX for 2 weeks. Representative images of cells stained with crystal violet are shown **(left)**. The colony number was quantified by ImageJ **(right)**, and results relative to vector are shown. Data are mean ± SEM. *n* = 3. **p < 0.01.

We then asked whether CSAG2 mediates the promotive effect of CPEB4 on paclitaxel resistance in ovarian cancer cells. CSAG2 was silenced by siRNA in KOV3 (S) cells overexpressing CPEB4 (Figure 5A), and these manipulations did not *per se* affect cell proliferation or survival under untreated condition (Figure 5B). On the other side, CSAG2 silencing significantly attenuated the survival advantage conferred by CPEB4 overexpression when treated with paclitaxel, although did not completely reduce to the extent of two vector groups (Figure 5C), at any rate, suggesting that CSAG2 knockdown attenuates CPEB4-mediated paclitaxel resistance in ovarian cancer cells. Notably, this effect of CSAG2 seems contextual, since CSAG2 silencing did not apparently affect paclitaxel resistance in vector groups (Figure 5C). Consistently, similar tendency was obtained when examining the effect of CSAG2 knockdown on CPEB4-mediated advantages for colony formation (Figure 5D), further strengthening the notion that CSAG2 at least in part contributes to CPEB4-mediated paclitaxel resistance in ovarian cancer.

## Discussion

Understanding the mechanisms of paclitaxel resistance is of great importance for developing effective therapeutic strategies to treat paclitaxel-resistant ovarian cancer. Our study provides biological basis and preclinical relevance that likely support a novel mechanism underpinning the paclitaxel resistance in ovarian cancer *in vitro*, in which CEPB4 is connected to the enhancement of paclitaxel resistance by its translational control of inducing CSAG2 expression, and thereby, uncovering the promotive function of CEPB4/CSAG2 axis in paclitaxel resistance in ovarian cancer. Deduced from our findings, it is possible that ovarian cancer cells may develop paclitaxel resistance through upregulating CEPB4 and thereafter inducing translational expression of CSAG2. Presumably, the higher levels of CEPB4 and CSAG2 this population of paclitaxel-resistant cells have, the more resistant these cells may respond to paclitaxel chemotherapy. If this is indeed the case and also applicable for paclitaxel resistance acquired by ovarian cancer after paclitaxel chemotherapy, the therapeutic strategy targeting CEPB4/CSAG2 axis, such as genetically reducing their expression or chemically inhibiting their activities, may thus be of potential clinical benefits for reducing paclitaxel resistance and enhancing the cytotoxic response of ovarian cancer to paclitaxel treatment.

The key aspect of the above proposition appears to rely on the activation of CEPB4 expression in ovarian cancer cells. In this study, in addition to paclitaxel-resistant ovarian cancer cell lines, SKOV3 and CaOV3, we also found that CPEB4 was upregulated at transcript and protein levels in recurrent ovarian tumors after treatment of paclitaxel-based chemotherapy, suggesting a molecular mechanism that transcriptionally regulates its expression induction is announced in ovarian cancer in response to paclitaxel. However, cautions are needed here when drawing a further conclusion about its clinical relevance, because the recurrent ovarian tumors were obtained from patients treated with cisplatin and paclitaxel combination chemotherapy. To date, although no reports have associated cisplatin with paclitaxel resistance in ovarian cancer, its possible effect can not be easily excluded. Therefore, to better relate CEPB4 upregulation to paclitaxel resistance, examining CEPB4 expression in recurrent ovarian tumors receiving paclitaxel monotherapy may provide a more direct evidence to demonstrate the specificity of this phenotype. Alternatively, at an opposite direction, future studies investigating whether CEPB4 responds to cisplatin treatment could also aid the estimation of its effect on paclitaxel.

On the other hand, however, the appearance of CEPB4 upregulation in ovarian cancer cells treated with paclitaxel seems to take a relatively long period of time, since CPEB4 upregulation was not observed as an acute responding mechanism, at least within 72 h observed in our study, to paclitaxel exposure. In other words, certain responsive mechanisms occurring at later phase may be responsible for its induction. However, at present, much less is known concerning the mechanisms controlling CEPB4 transcription.

CEPB4 is an RNA binding protein that controls meiotic mRNA cytoplasmic polyadenylation and translation (Novoa et al., 2010). Its role in cancer is barely investigated, until recently its overexpression in pancreatic ductal adenocarcinomas and glioblastomas was reported, where it could support tumor growth, vascularization and invasion by translational activation of mRNAs including the tissue plasminogen activator (Ortiz-Zapater et al., 2011). In addition, its upregulated expression in human glioma (Hu et al., 2015), breast cancer (Sun et al., 2015), astrocytic tumor (Chen W. et al., 2016) hepatocellular carcinoma (Tsai et al., 2016), colorectal carcinoma (Cortes-Guiral et al., 2017), has been correlated with disease progression and poor prognosis. These studies suggest a pro-oncogenic role of CEPB4 in tumorigenesis. One previous study has shown that the enhancer region of CEPB4 gene binds with transcriptional factors Gata1 and Tal1, and during terminal erythroid differentiation, Gata1 and Tal1 induce its transcription (Hu et al., 2014). Moreover, CPEB4 gene could also be directed by p53 transcriptional targets (Zaccara et al., 2014). Whether these regulatory circuitries are responsible for CEPB4 upregulation in paclitaxel-resistant ovarian cancer cells and recurrent ovarian tumors needs intensive investigations. In functional studies, we found that CPEB4 promoted paclitaxel resistance in ovarian cancer cells *in vitro*. In despite of the largely unclear role of CPEB4 in ovarian cancer progression, our study reveals its connection to paclitaxel resistance in ovarian cancer.

By using RIP assay, followed by validation, we demonstrate that the mRNA of CSAG2, one protein closely related to paclitaxel resistance in ovarian cancer, binds with CEPB4. Although CSAG2 has been associated with paclitaxel resistance in ovarian cancer *in vitro* and in clinic study (Materna et al., 2007), its specific functions remain elusive and contradictory (Duan et al., 1999). In this study, we validate CSAG2 as a paclitaxel-resistant protein. CSAG2 is more polyadenylated and translated by CPEB4 in paclitaxel-resistant ovarian cancer cells and recurrent ovarian tumors, and this post-transcriptional regulation of CSAG2 by CPEB4 has a causal link to paclitaxel resistance, because CSAG2 knockdown attenuates CPEB4-mediated paclitaxel resistance in ovarian cancer cells. Nevertheless, the unsolved question is how CSAG2 exerts resistance response to paclitaxel treatment. In a latest study, CSAG2 was demonstrated necessary for proliferation and tumorigenesis *in vivo*, and CSAG2-stimulated SIRT1 activity to enhance p53 deacetylation was shown to inhibit p53 transcriptional activity, leading to improved cell survival under genotoxic stress (Yang and Potts, 2020). We speculate this regulatory circuit may be one of the mechanisms through which CSAG2 promotes paclitaxel resistance in ovarian cancer. Another critical issue needs to be addressed is whether CPEB4-regulated paclitaxel resistance *via* CSAG2 in ovarian cancer can be reproduced with *in vivo* scenarios. Elucidating these issues would not only help us to deeply understand paclitaxel resistance in ovarian cancer, but also develop a rational therapeutic treatment targeting active CEPB4/CSAG2 axis in paclitaxel-resistant ovarian cancer.

## Data Availability Statement

The raw data supporting the conclusions of this article will be made available by the authors, without undue reservation.

## Ethics Statement

The studies involving human participants were reviewed and approved by Ethics Committee of Medical College of Northwest Minzu University. The patients/participants provided their written informed consent to participate in this study.

## Author Contributions

Conception and design: JZ and YZ; Provision of study materials or patients: XM; Collection and assembly of data: RH; Data analysis and interpretation: XX; Manuscript writing and final approval of manuscript: All authors.

## Funding

This work was supported by Fundamental Research Funds for the Central Universities (Grant No. 31920200030 and 31920190210), the Science and Technology Development Project of Lanzhou city Chengguan District (No. 2018-1-10) and the National Natural Science Foundation of China (NSFC, No. 81860716).

## Conflict of Interest

The authors declare that the research was conducted in the absence of any commercial or financial relationships that could be construed as a potential conflict of interest.
