# Supplementary material for: CPEB4-Promoted Paclitaxel Resistance in Ovarian Cancer In Vitro Relies on Translational Regulation of CSAG2
Source: Front Pharmacol. 2021 Jan 13;11:600994. doi: 10.3389/fphar.2020.600994 (PMC7838559; doi:10.3389/fphar.2020.600994)
Supplement: Supplementary file 1 [file table1.docx]

| Supplementary table 1. Clinicopathologic characteristics of patients | | | | | |
| --- | --- | --- | --- | --- | --- |
|  | Patients | |  |  |  |
| Characteristic | No. (N=18) | % |  |  |  |
| Age | | |  |  |  |
| ≤60 | 5 | 27.8 |  |  |  |
| > 60 | 13 | 72.2 |  |  |  |
| Histology | | |  |  |  |
| Serous | 11 | 61.1 |  |  |  |
| Mucinous | 2 | 11.1 |  |  |  |
| Endometrioid | 3 | 16.7 |  |  |  |
| others | 2 | 11.1 |  |  |  |
| FIGO stage | | |  |  |  |
| Ⅲ | 11 | 61.1 |  |  |  |
| Ⅳ | 7 | 38.9 |  |  |  |
| Grade | | |  |  |  |
| G1 | 2 | 11.1 |  |  |  |
| G2,G3 | 16 | 88.9 |  |  |  |
| Abbreviation: FIGO, Federation of Gynecology and Obstetrics. | | | | |  |
|  |  |  |  |  |  |
